# Update in Heart Rhythm Abnormalities and Indications for Pacemaker After Transcatheter Aortic Valve Implantation

**DOI:** 10.21470/1678-9741-2017-0206

**Published:** 2018

**Authors:** Marina Saadi, Ana Paula Tagliari, Luiz Cláudio Danzmann, Eduardo Bartholomay, Adriano Nunes Kochi, Eduardo Keller Saadi

**Affiliations:** 1 Universidade Luterana do Brasil (ULBRA), Canoas, RS, Brazil.; 2 Department of Cardiovascular Surgery, Hospital de Clínicas de Porto Alegre (HCPA), Universidade Federal do Rio Grande do Sul (UFRGS), Porto Alegre, RS, Brazil.; 3 Department of Cardiology, Universidade Luterana do Brasil (ULBRA), Canoas, RS, Brazil.; 4 Department of Cardiology, Hospital de Clínicas de Porto Alegre (HCPA), Universidade Federal do Rio Grande do Sul (UFRGS), Porto Alegre, RS, Brazil.

**Keywords:** Aortic Valve Stenosis, Transcatheter Aortic Valve Implantation, Arrhythmias, Cardiac, Pacemaker, Artificial

## Abstract

Objective: Rhythm abnormalities following transcatheter aortic valve implantation (TAVI) and indications for permanent pacemaker implantation (PPI) were reviewed, which aren't well established in the current guidelines. New left bundle branch block and atrioventricular block are the most common electrocardiographic changes after TAVI. PPI incidence ranges from 9-42% for self-expandable and 2.5-11.5% for balloon expandable devices. Not only anatomical variations in conduction system have an important role in conduction disorders, but different valve characteristics and their relationship with cardiac structures as well. Previous right bundle branch block has been confirmed as one of the most significant predictors for PPI.

**Table:** 

Abbreviations, acronyms & symbols	
AoS	= Aortic valve stenosis
AV	= Atrioventricular
AVB	= Atrioventricular block
CI	= Confidence interval
ECG	= Electrocardiogram
EPS	= Electrophysiological study
HR	= Hazard ratio
LAHB	= Left anterior hemiblock
LBBB	= Left bundle branch block
LVOT	= Left ventricular outflow tract
OR	= Odds ratio
PPI	= Permanent pacemaker implantation
RBBB	= Right bundle branch block
RR	= Relative risk
SAVR	= Surgical aortic valve replacement
TAVI	= Transcatheter aortic valve implantation

## INTRODUCTION

Increased life expectancy has contributed to making cardiac valve diseases more frequent and relevant^[^^1^^]^.

Aortic valve stenosis (AoS), the most common cardiac valve disease, affects approximately 3% of the population older than 75 years^[^^2^^]^ and, usually, remains silent for many years.

However, once symptoms are present, survival decreases dramatically^[^^3^^,^^4^^]^, being surgical treatment the therapy of choice^[^^5^^]^.

Since the first clinical report in 2002, transcatheter aortic valve implantation (TAVI) has emerged as a valuable, less invasive and safe therapeutic alternative in patients with symptomatic severe AoS^[^^6^^]^.

Currently, TAVI is considered the gold standard for high-risk patients, the only option for inoperable ones, and, recently, non-inferior to conventional surgery in intermediate risk patients^[^^7^^-^^9^^]^.

On the other hand, this is not a risk-free procedure, with atrioventricular (AV) conduction disturbances being a common complication^[^^10^^]^.

In this context, understanding post-procedural conduction disturbances' mechanisms and rates is an extremely current and relevant issue.

This study aims to review rhythm abnormalities after TAVI, besides the permanent pacemaker implantation (PPI) indications, which are not well established by the current guidelines.

### Rhythm Abnormalities

Bradyarrhythmias are not uncommon after cardiac surgery, TAVI and heart transplantation. According to the 2013 European Society of Cardiology guidelines on cardiac pacing and cardiac resynchronization therapy^[^^11^^]^, complete atrioventricular block (AVB) may occur in 1-4% of cardiac surgeries, 8% of repeat valve surgeries and up to 20-24% of interventions for calcified AoS or tricuspid valve replacement.

Following TAVI, new left bundle branch block (LBBB) and AVB are the main electrocardiographic changes and the consequent need for a permanent pacemaker is the most expensive short-term adverse event^[^^12^^]^.

The incidence of post-procedural conduction disturbances varies among studies, being 20% in complete AVB, 7% to 83% in LBBB, 2% in right bundle branch block (RBBB) and 2% in left anterior hemiblock (LAHB)^[^^13^^-^^16^^]^.

By Urena et al.^[^^13^^]^, 37.7% of these new LBBB are resolved before hospital discharge and 57% in 6-12-month follow-up period.

The PARTNER trial analysis showed that, although new LBBB has not been associated with increased 1-year mortality, it was associated with a higher in-hospital pacemaker indication [8.3% *versus* 2.8%; *P*=0.005] and from discharge to one year (4.7% *vs*. 1.5%; *P*=0.01), as well as failure to improve left ventricular ejection fraction (52.8 *vs.* 58.1%; *P*<0.001)^[^^17^^]^.

In contrast, another study showed new LBBB as an independent predictor of all-cause mortality [hazard ratio (HR) 1.54; 95% confidence interval (CI) 1.12 - 2.10]. Cardiovascular mortality rate was 9.4% for patients without TAVI-induced LBBB and 18% for new LBBB^[^^15^^]^.

Similarly, for Makki et al.^[^^18^^]^, PPI has been associated with increased short- and long-term mortality, which may represent inherent conduction abnormality or pacemaker operation morbidity.

### PPI Indications

Pacemaker implantation after TAVI has had the same recommendations as non-surgical patients with persistent bradycardia, such as symptomatic bradycardia, complete AVB, type II second-degree AVB, new LBBB in combination with infra-hisian conduction delay (HV interval ≥70 ms or second or third-degree Hiss-Purkinje block)^[^^11^^]^.

The 2013 European Society of Cardiology guideline considers indication class I, level C "high degree or complete AVB after cardiac surgery and TAVI, with a period of clinical observation up to 7 days (Table 1). In case of complete AVB with a low rate of escape rhythm, this observation period can be shortened since the resolution is unlikely"^[^^11^^]^.

**Table 1:** Recommendation for pacing after transcatheter aortic valve implantation^[^^11^^]^.

Recommendation	Class of recommendation	Level
I) High degree or complete AV block after TAVI	I	C

In the post-TAVI phase, the management depends on the prosthesis implanted. For instance, early removal of the temporary pacing lead seems to be safe after the balloon expandable Edwards Sapien® prosthesis, in the absence of intraprocedural advanced degree blocks. Then, a 24-48 hour electrocardiographic monitoring followed by a daily 12-leads electrocardiogram (ECG) until the discharge is recommended. On the contrary, when a self-expandable CoreValve® prosthesis is chosen, a 48-72 hour monitoring before removal of the temporary pacing lead might be recommended even when the ECG early after TAVI is normal^[^^19^^]^.

QRS duration is another factor that may assist the decision about early or late pacing lead removal. According to a Takahashi's et al.^[^^20^^]^ study, in which patients with QRS duration <120 ms were submitted to early pacing lead removal and those with QRS ≥120 ms to late removal, no PPI was indicated in the first group, while 39% of the latter developed a delayed AVB (*P*=0.0001).

Taking into account that TAVI is routinely performed under heparin anticoagulation, which potentially may lead to an increased bleeding rate for PPI immediately after TAVI, Schwerg et al.^[^^21^^]^ evaluated patients who have undergone PPI implantation on the same day of TAVI implant (group A) and patients in whom the PPI was performed at least 1 day after TAVI (group B) (3.8±4.5 days). In this study, procedure times, fluid loss via drainage systems, and drainage times were neither significantly different between the groups nor when compared to a historical group.

### PPI Risk Factors

Many factors have been identified for PPI requirement after TAVI, mainly related to baseline conductions defects, device selection and anatomical characteristics^[^^22^^]^. Thus, a careful pre-TAVI screening is overwhelming important^[^^19^^]^.

Advanced age, permanent atrial fibrillation, use of digoxin, larger or oversized prosthesis^[^^23^^,^^24^^]^, for example, are considered clinical predictors for new LBBB post-TAVI, while left heart axis, lower mean heart rate, and prolonged PQ and QRS times are considered electrocardiographic parameters for severe cardiac conduction defects requiring PPI in patients with new LBBB^[^^24^^]^.

During the procedure predilatation and valve deployment are the most critical steps for conduction disorders development^[^^25^^,^^26^^]^.

Regarding the available devices, the differences in shape, height of the frame, depth of implantation and different physical properties account for the different PPI incidences observed^[^^19^^]^.

A meta-analysis published in 2014 with 41 studies comprising 11210 post-TAVI patients, showed an unadjusted 2.5-fold higher risk for CoreValve® (28%) compared to Sapien® (6%). The risk was increased for men [relative risk (RR) 1.23; *P*<0.01]; previous first-degree AVB (RR 1.52; *P*<0.01), LAHB (RR 1.62; *P*<0.01) or RBBB (RR 2.89; *P*<0.01) and intraprocedural AVB (RR 3.49; *P*<0.01)^[^^27^^]^.

In a more recent study, the combination of pre-TAVI prolonged PR interval (>220 ms) and increased QRS duration (>120 ms) reached a positive predictive value for PPI of 80%, suggesting to use such parameters as periprocedural PPI markers^[^^28^^]^.

Analyzing national data, the Brazilian Registry of 418 patients with severe AoS who had undergone TAVI demonstrated a 30-day PPI incidence of 25.2%. On multivariable analysis only CoreValve® *vs.* Sapien XT® [odds ratio (OR) 4.24, 95% CI 1.56 - 11.49; *P*=0.005], baseline RBBB (OR 4.41, 95% CI 2.20 - 8.82; *P*<0.001), and balloon pre-dilatation (OR 1.75, 95% CI 1.02 - 3.02; *P*=0.04) were independent PPI predictors^[^^29^^]^.

Supporting these data, solely baseline RBBB and deep valve implantation were found as predictors of high-degree AVB and PPI requirement in a separate multivariable analysis of a study published in 2011 in the American Journal of Cardiology^[^^30^^]^.

In terms of potential tools for AVB risk stratification in the electrophysiological study (EPS), a cohort led by Kostoupoulou et al.^[^^16^^]^ analyzed 30 patients who underwent EPS, 25 of these had a second EPS after 48h. Delta-HV did not show to be a risk factor and baseline HV interval did only in univariate analysis.

Opposing these findings, physicians from the Montreal Heart Institute followed 75 patients who had undergone EPS before and after TAVI. In multivariate analysis, the delta-HV interval was independently associated with AVB development and its sensitivity and specificity for predicting AVB were, respectively, 100% and 84.4% for a delta-HV interval ≥13ms^[^^31^^]^.

Considering the left ventricular outflow tract (LVOT) in terms of amount of calcium, perimeter and device size relative to LVOT, as well as the degree of valve protrusion into the LVOT, once these factors could affect the underlying conduction system, Rodríguez-Olivares et al.^[^^32^^]^ performed a study where computed tomography was used to assess LVOT. By multivariate analysis RBBB at baseline (OR 2.9, 95% CI 1.2 - 6.9; *P*=0.014), next generation valves (OR 2.1, 95% IC 1.0-4.5; *P*=0.048), depth of implantation (OR 1.2 per 1 mm increment, 95% IC 1.1-1.3; *P*<0.001) and LVOT sizing (OR per 1% increment 1.0, 95% IC 1.0-1.06; *P*=0.022) were predictors of PPI.

### Heart Block Mechanisms after TAVI

By Young Lee et al.^[^^10^^]^ inter-individual variation in the penetrating bundle length and depth of septal penetration and variation in the location of the proximal portion of left bundle determine how susceptible these structures are to injury during TAVI.

While conduction abnormalities in surgical aortic valve replacement (SARV) are attributed to the surgical method - suturing along the sewing ring near the membranous septum, removal of the native aortic valve and edema - the susceptibility to AVB in TAVI is more specific.

In CoreValve®, the AVB risk is partly due to the valve design and the potential for a deeper valve implantation into the LVOT^[^^10^^]^.

Kammler et al.^[^^33^^]^ identified that the mean distance from the annular margin of the non-coronary cusp to the ventricular end of the prosthesis was significantly longer in patients with pacemaker requirement (9.7±4.1 mm *vs*. 6.3±3.4 mm; *P*=0.0017). A cut-off value of 6 mm predicted this need with a sensitivity of 89% and specificity of 40%.

Nuis et al.^[^^25^^]^ also reported a direct relationship between the balloon/annulus ratio during balloon valvuloplasty and the development of new conduction disturbance, with a balloon/annulus ratio close to 1.0 as an acceptable compromise to avoid it.

### PPI Rates

PPI after TAVI incidence has been reported ranging from 9% to 42% for self-expandable valves (CoreValve®) and from 2.5% to 11.5% for balloon expandable valves (Edwards Sapien®)^[^^34^^]^.

In the Partner 2 trial, where the Sapien XT® system was used in patients with severe AoS and intermediate surgical risk, the 30-day PPI rate was 8.5%^[^^7^^]^.

Comparing two generations of self-expanding repositionable valves, the PPI rates were 25.5% for CoreValve® and 26.7% for the new generation Evolut R®^[^^8^^]^.

In the CHOICE trial, a lower 30-day pos-procedural PPI need was observed in the balloon-expandable group (Sapien XT®) when compared to the self-expandable group (CoreValve®) (17.3% *vs.* 37.6%, RR 0.46, 95% CI 0.28 - 0.74). Besides, cardiovascular mortality within 30 days was 4.1% in the first and 4.3% in the second group (RR 0.97, 95% CI 0.29 - 3.25; *P*=0.99)^[^^35^^]^.

Another study comparing second generation devices - Direct Flow Medical®, Lotus®, Evolut R® or Sapien 3® (2G) to first generation - Sapien XT®, CoreValve® (1G), showed no differences regarding PPI requirement (6.5% *vs.* 7.8%; *P*=0.46); but patients treated with 1G devices suffered more 30-day adverse events (free of adverse events 75.3% *vs.* 88.8%, HR 2.4; 95% CI 1.4 - 4.0; *P*=0.01)^[^^36^^]^.

According to access routes, The German aortic valve registry (GARY), the biggest registry about TAVI, showed that 1-year pacemaker need was 26.2% in transvascular approach and 14.1% in transapical^[^^37^^]^.

Finally, in a meta-analysis published in 2016, which compared TAVI with conventional SVAR in low and intermediate risk patients, a higher PPI rate after TAVI was observed when compared to SAVR (21.6% *vs.* 7.5%, OR 7.4, 95% CI 1.98 - 8.34; *P*<0.001)^[^^38^^]^. The same finding has not been seen with sutureless devices, which have a PPI incidence similar to TAVI (5-17%)^[^^39^^-^^42^^]^.

## CONCLUSION

Not only anatomical variations in each patient conduction system may have an important role in the conduction disorders prevalence after TAVI, as well as the different valve implanted and its characteristics in terms of diameter and relationship with other cardiac structures. Self-expandable devices are related to a higher incidence of PPI need than balloon-expandable valves.

Some baseline features, like RBBB, have been confirmed as significant predictors of PPI need. Moreover, new LBBB, which most often occur in the first 48 hours after TAVI, may also be a PPI risk factor.

Further studies may be necessary for better understanding of rhythm abnormalities and to establish the permanent pacemaker implantation criteria after TAVI.

**Table:** 

Authors' roles & responsibilities	
MS	Substantial contributions to the conception or design of the work; or the acquisition, analysis, or interpretation of data for the work; drafting the work or revising it critically for important intellectual content; final approval of the version to be published
APT	Substantial contributions to the conception or design of the work; or the acquisition, analysis, or interpretation of data for the work; drafting the work or revising it critically for important intellectual content; final approval of the version to be published
LCD	Substantial contributions to the conception or design of the work; or the acquisition, analysis, or interpretation of data for the work; drafting the work or revising it critically for important intellectual content; final approval of the version to be published
EB	Substantial contributions to the conception or design of the work; or the acquisition, analysis, or interpretation of data for the work; drafting the work or revising it critically for important intellectual content; final approval of the version to be published
ANK	Substantial contributions to the conception or design of the work; or the acquisition, analysis, or interpretation of data for the work; drafting the work or revising it critically for important intellectual content; final approval of the version to be published
EKS	Substantial contributions to the conception or design of the work; or the acquisition, analysis, or interpretation of data for the work; drafting the work or revising it critically for important intellectual content; final approval of the version to be published
